# Examination of the Urine Discharged by Patients Affected with Diabetes

**Published:** 1804-04-01

**Authors:** 


### Examination of the Urine discharged by Patients affected with Diabetes. By Messrs. Nicholas and Guedeville, of Caen.

### *Physical Properties of the Diabetical Urine.*

1. It has no smell, but a saccharine taste. 2. It is turbid and whitish.

### *Analysis by Means of Reagency.*

1. It changes the blue colour of the tinctura heliotropii into violet blue. 2. Nitrat of silver occasions a caseous precipitation, but soon after this becomes violet, and the liquor is rendered clear, of a pale yellow colour, and acquires no smell. 3. Lime water renders this urine instantly turbid, disengaging a slight smell of ammonia, and some time after a white flaky precipitate is formed, consisting of phosphat of lime, after which the liquor becomes as clear as the purest water. 4. Concentrated sulphuric acid changes the colour of diabetical urine into a rose colour, but occasions no considerable precipitation. 5. Sulphat of mercury troubles this urine and makes a reddish precipitate, which is partly kept swimming in the liquor. 6. Kali occasions a far less precipitation and disengagement of ammonia, than it does in healthy urine. 7. Muriat of lead imparts a milky colour to it; and when the precipitate has subsided, the liquor remains quite clear.

### *Analysis by Evaporation.*

On exposing diabetical urine for about six weeks to the accession of air at a temperature of from 10 to 12°, it became turbid the first six days, depositing a white flaky substance which proved to be albuminous matter. The clear liquor had an acid smell and taste, and strongly reddened the blue vegetable colour. The acid urine perfectly united with a solution of carbonated kali without much effervescence, and on having filtrated and evaporated the liquor, acetite of kali mixed with a small quantity of phosphat of kali was obtained. On evaporating four pounds of diabetical urine by a gentle heat, four ounces and a half of extract, of the consistency of honey, were obtained, which had a brown colour, and the smell of burned sugar. Ten parts of this extract being mixed with four parts of muriat of barytes and one part of charcoal powder, were well dried, and submitted to distillation in a retort, tort, but not any trace of ammonia or phosphorus could be perceived, nothing except carbonated hydrogen gas and a black oil of a disagreeable smell was disengaged. On adding four ounces of alcohol of 35° to one ounce and two drachms of inspissated urine, a yellow clear liquor was obtained, which however, twenty-four hours after, deposited a flaky greyish matter, weighing twenty grains, of a saline and rather saccharine taste. Lime water disengaged a slight smell of ammonia from it, and sulphuric acid blackened it, which experiment was attended with disengagement of sulphurous acid; all which intimates the presence of albuminous matter in the residuum. Part of the above mentioned extract, distilled with nitric acid, yielded oxalic acid and a small portion of phosphorus acid, and during this operation a great quantity of nitrous gas was disengaged. Four ounces and a half of the extract were diluted with one pound of distilled water, to which was added half an ounce of dry yeast. This mixture being exposed to a temperature of 15°, twenty-four hours after, the liquor began to ferment, whereby a great quantity of carbonic gas was disengaged. When the fermentation was finished, the author submitted the liquor to a distillation, by which ten ounces of a weak spirituous liquor were obtained, the smell of which was by no means agreeable. By rectifying, it yielded four ounces and two drachms of alcohol of 32°, which however retained the same disagreeable smell.

In order to separate the sugar from this urine, four ounces of the inspissated urine were diluted with one pound of water, and boiled, with the addition of one ounce of blood. After the liquor had been suffered to settle it was separated from the sediment, and clarified by the white of an egg. It was then evaporated to the consistency of a syrup, and exposed to the atmosphere; eight days after, small brownish crystals were perceived, which were neither so regular nor so hard as those of sugarcandy. When the liquor had been poured into an earthen vessel, it became dry five days after, resembling in form and colour common powdered sugar. Some experiments made on this substance proved it to be only saccharine mucilage, as it was decomposed by caustic kali.

The results of these different experiments are the following: 1. The urine discharged by persons affected with diabetes contains neither urée, uric acid, nor benzoic acid. 2. Ammonia and phosphats are present, but in a very small proportion. 3. It is capable of undergoing the vinous and acid fermentation. 4. It yields accordingly alcohol and acetic acid. 5. It contains saccharine mucilage.

 The

The authors likewise examined the blood of diabetical patients; and from their experiments it appears, that the blood of such persons contains no saccharine mucilage; that the fibrous matter is present in a smaller proportion; and the serum abounds more than in a healthy state.

The saccharine mucilage is composed of oxygen, hydrogen, and carbon; whereas the urée contains a great proportion of azote, the absence of which occasions the generation of saccharine matter, and the diminution of the fibrous substance. When a due proportion of the azote is restored, the urée will again be generated and the fibrous substance augmented. For the cure of diabetes it is therefore advisable, first, to order a diet of substances which contain much azote, namely an animal diet; and, secondly, to prescribe those remedies which are chiefly composed of azote and phosphoric acid, as ammonia internally as well as externally; phosphats and phosphorous acid; and, finally, to remove the morbid state which prevents the due animalisation of the aliments.

